# Value-driven attentional capture enhances distractor representations in early visual cortex

**DOI:** 10.1371/journal.pbio.3000186

**Published:** 2019-08-09

**Authors:** Sirawaj Itthipuripat, Vy A. Vo, Thomas C. Sprague, John T. Serences

**Affiliations:** 1 Learning Institute, King Mongkut’s University of Technology Thonburi, Bangkok, Thailand; 2 Futuristic Research in Enigmatic Aesthetics Knowledge Laboratory, King Mongkut’s University of Technology Thonburi, Bangkok, Thailand; 3 Department of Psychology and Center for Integrative and Cognitive Neuroscience, Vanderbilt University, Nashville, Tennessee, United States of America; 4 Neurosciences Graduate Program, University of California San Diego, La Jolla, California, United States of America; 5 Department of Psychology, New York University, New York, New York, United States of America; 6 Department of Psychological and Brain Sciences, University of California Santa Barbara, Santa Barbara, California, United States of America; 7 Department of Psychology and Kavli Foundation for the Brain and Mind, University of California San Diego, La Jolla, California, United States of America; Vanderbilt University, UNITED STATES

## Abstract

When a behaviorally relevant stimulus has been previously associated with reward, behavioral responses are faster and more accurate compared to equally relevant but less valuable stimuli. Conversely, task-irrelevant stimuli that were previously associated with a high reward can capture attention and distract processing away from relevant stimuli (e.g., seeing a chocolate bar in the pantry when you are looking for a nice, healthy apple). Although increasing the value of task-relevant stimuli systematically up-regulates neural responses in early visual cortex to facilitate information processing, it is not clear whether the value of task-irrelevant distractors influences behavior via competition in early visual cortex or via competition at later stages of decision-making and response selection. Here, we measured functional magnetic resonance imaging (fMRI) in human visual cortex while subjects performed a value-based learning task, and we applied a multivariate inverted encoding model (IEM) to assess the fidelity of distractor representations in early visual cortex. We found that the fidelity of neural representations related to task-irrelevant distractors increased when the distractors were previously associated with a high reward. This finding suggests that value-driven attentional capture begins with sensory modulations of distractor representations in early areas of visual cortex.

## Abstract 2

Task-irrelevant distractors can automatically capture attention when they were previously associated with a high reward. This study shows that these high-valued distractors increased the fidelity of neural representations in retinotopically organized regions of visual cortex, showing that value-driven attentional capture begins with early sensory modulations of distractor representations in human visual system.

## Introduction

In most real-world situations, stimuli that are visually salient—such as a camera flash in a theater or a green object in a sea of red—automatically capture attention [1–4]. Likewise, distractors that are distinguished only by their value, not their visual salience, also capture visual attention—even on occasions when high-valued distractors are unactionable and irrelevant to current behavioral goals (e.g., seeing a piece of cake on another table, but your waiter tells you it is no longer available) [5–10]. In the laboratory, the value associated with an irrelevant distractor interferes with the processing of task-relevant visual information, resulting in increased response times (RTs) and sometimes reduced accuracy in a variety of tasks ranging from simple visual discrimination to more complex scenarios in which the value of multiple competing items must be compared [5–8,10–17]. Importantly, these behavioral effects of value-based attentional capture are underexpressed and overexpressed in patients with attention-deficit hyperactivity disorder and addiction, respectively [18,19]. Although previous work has shown that the value of task-relevant visual information increases neural activity in areas of early visual cortex [20–26], it is unclear how the learned value of irrelevant distractors modulates cortical responses in these cortical regions. Thus, here we aimed to examine the involvement of early visual cortex in supporting attentional control guided by the learned value associated with task-irrelevant distractors.

Several theories of reinforcement learning have emphasized the essential role of reward in prioritizing sensory information [27–34]. For example, stimuli associated with high reward are thought to better compete for sensory representations such that those stimuli become visually salient and automatically capture attention, even though these highly rewarded stimuli might be physically nonsalient [7]. Based on this previous work, some have proposed that dopaminergic neurons in the midbrain and in the ventral striatum relay value-related signals to cortical regions within the early visual cortex, resulting in the potentiation of visual representations related to high-reward visual stimuli [35–37]. According to this proposal, we hypothesized that the learned value associated with irrelevant distractors should enhance distractor representations in early visual cortex and that this enhancement should be spatially restricted to the distractor locations, much like value-based modulations of task-relevant visual stimuli [23,25]. Alternatively, we might expect no value-based modulations or even a reduction in the response to distractors, as recent studies have shown that higher reward can lead to the suppression of distractor-related neural representations (see more details in the Discussion section; also [38–41]).

To test these alternative accounts, we recruited human participants to perform a value-based decision-making task and measured their brain activity in visual cortex using functional magnetic resonance imaging (fMRI). Subjects were required to select 1 of 2 task-relevant options while ignoring a third irrelevant and unactionable distractor that was rendered in a color that had been previously associated with a variable level of reward. We hypothesized that the previously assigned value of the distractor color would modulate evoked responses in early visual cortex and that this reward-based modulation would be specific to the spatial location of the distractor stimulus. To evaluate spatially selective modulations, we used an inverted encoding model (IEM) to reconstruct a representation of each stimulus using activation patterns of hemodynamic responses from retinotopically organized visual areas V1, V2, and V3. We chose this multivariate analysis over the univariate analysis of fMRI data because we were interested in examining how modulations of large-scale activation patterns across entire visual areas supported value-driven attention. The IEM method is useful here because it exploits modulations of all voxels in areas of early visual cortex, including attention-related increases and decreases in the hemodynamic response, as well as shifts in the position of voxel receptive fields [20,42–55]. We found that distractors previously associated with a high value slowed choice RTs. These high-value distractors were also represented with higher fidelity in extrastriate visual areas V2 and V3. Importantly, these value-based modulations of behavior and of neural representations depended on target selection history—these modulations were only observed when participants had previously selected and learned the value of irrelevant distractors. Together, these results suggest that the influence of high-value distractors on attentional capture begins with an early modulation of sensory responses and that this value-driven attentional capture occurs when participants have learned the value associated with the visual feature of the distractor.

## Results

### High-valued distractors automatically capture attention

**Fig 1:**
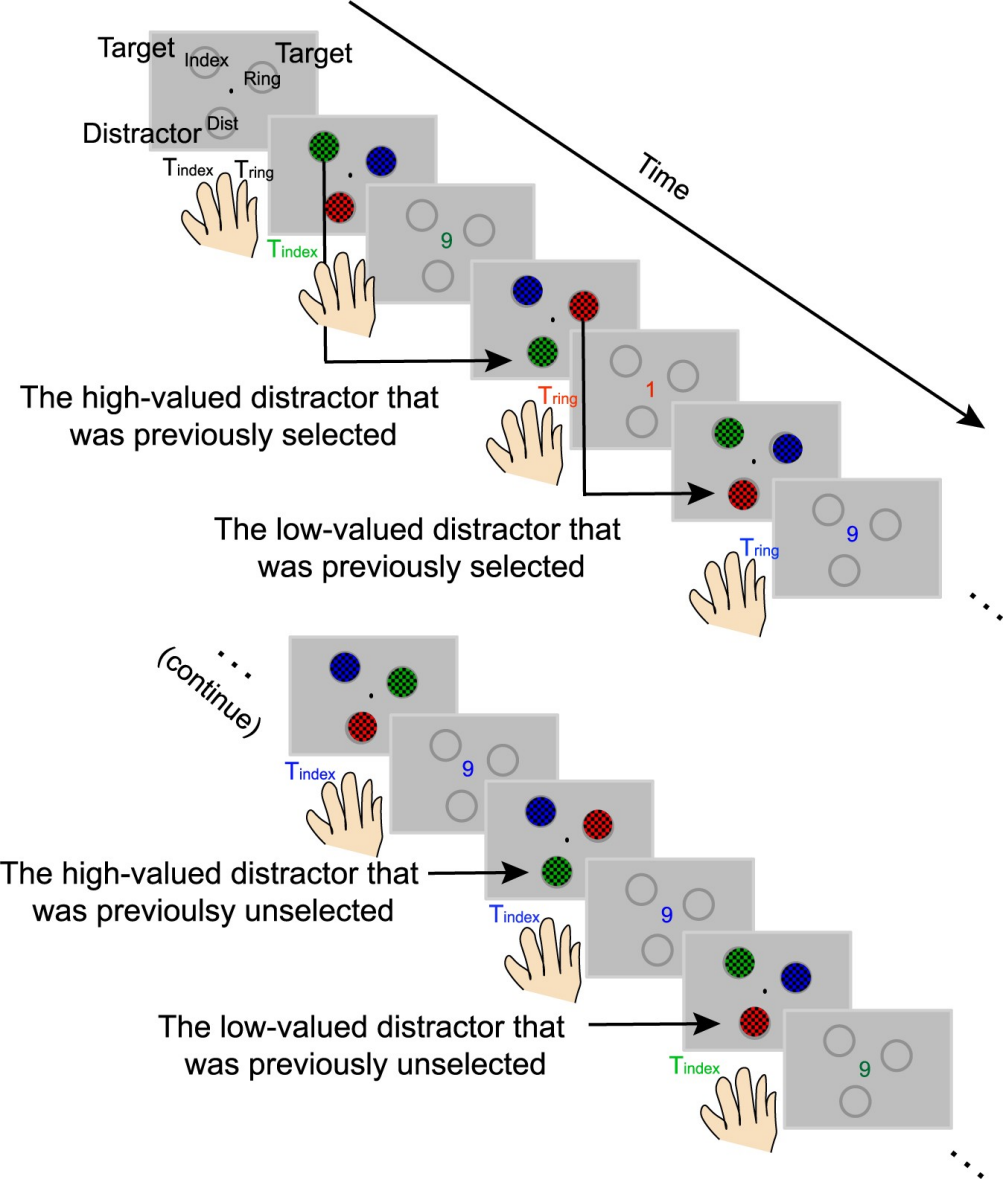
Value-based decision-making task. Participants selected 1 of the 2 target stimuli to learn values associated with their colors while ignoring a task-irrelevant distractor that could never be selected and was thus unactionable. Across trials, the colors of the targets and the distractor changed randomly so that the distractor color on a given trial could match the color of a previously selected target that yielded either a low or a high monetary reward (i.e., low- or high-valued distractor).

In the present study, we used fMRI to measure activity in retinotopically organized visual areas V1, V2, and V3 while human participants (*N* = 15) performed a 2-alternative value-based decision-making task with changing reward associations [6] (Fig 1). On each trial, 3 stimuli were presented, each rendered in a different color. Two of the stimuli were presented in fixed target locations, and subjects had to choose between them. The third stimulus, termed a “distractor,” was presented in another fixed location that subjects could never select. Participants learned that different rewards (1 or 9 cents) were associated with the colors of visual stimuli presented at the two target locations. Importantly, the distractor was not actionable and was thus completely irrelevant with respect to evaluating the relative value of the two possible targets. Across trials, the colors of the targets and the distractor changed randomly so that the distractor color on a given trial could match the color of a previously selected target that yielded either a low or a high monetary reward. Additionally, the pairings between color and reward changed across miniblocks of 8 trials so that values assigned to different colors could be counterbalanced. Thus, for behavioral and fMRI analyses, we sorted trials based on incentive values assigned to the colors of distractors (i.e., low- or high-valued distractor). The incentive value was always defined. However, a given color may not have been selected on previous trials. Therefore, the current value of the distractor was not always known to the participant. We thus used the selection of previous choices to examine the influence of learning reward contingencies on value-driven attention. To do so, we coded whether the distractor in a given trial was selected as a target in the previous 3 trials (i.e., selected or unselected; see Materials and methods). Note that we used the previous 3 trials in the main analysis because this data-sorting criterion yielded the most balanced number of trials across different experimental conditions (and we examine different sorting schemes in S2 Fig, detailed below).

**Fig 2:**
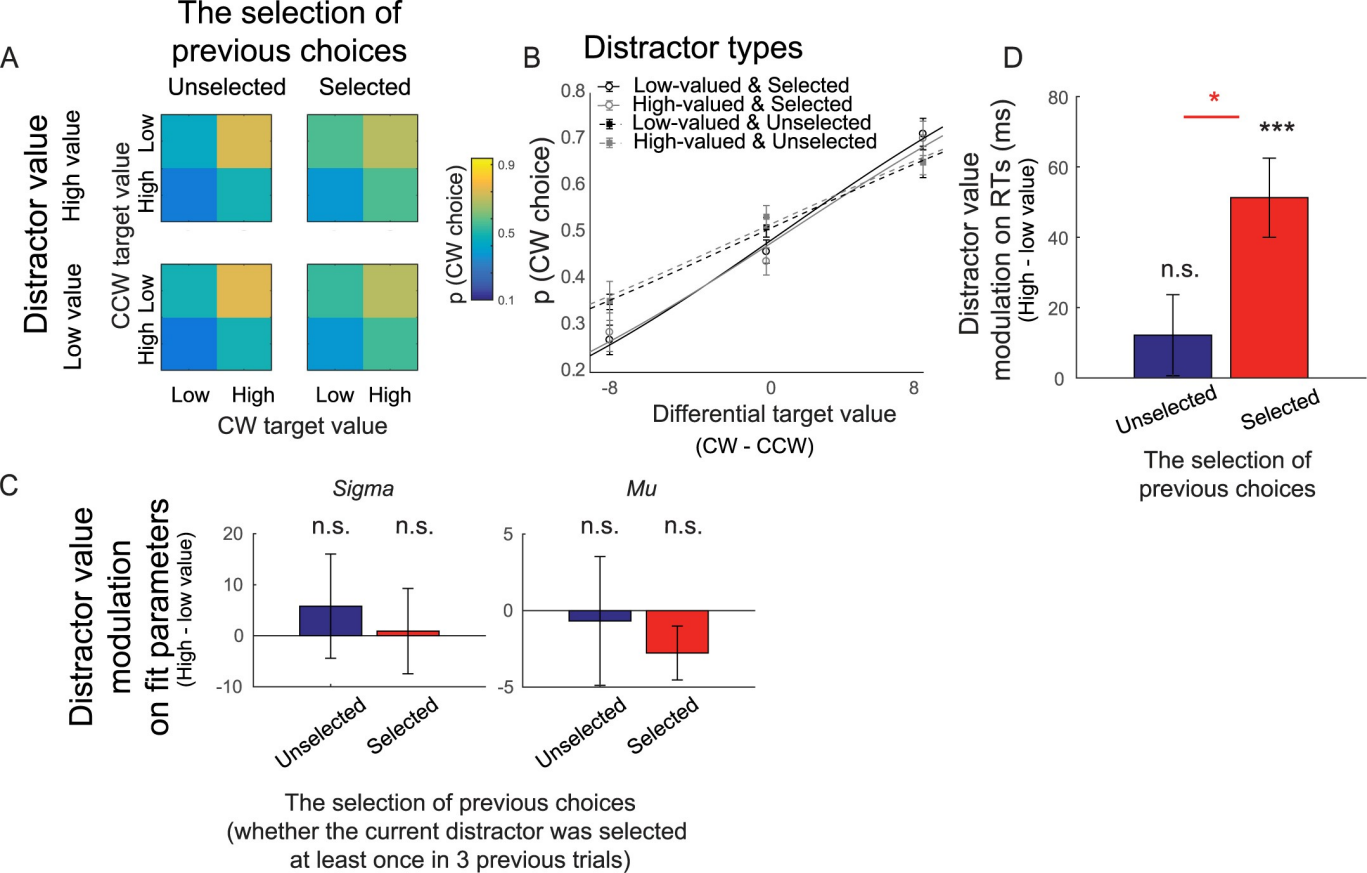
High-valued distractors increased RTs. (A) Choice preference for high-valued targets for different distractor types. CW and CCW targets are targets located clockwise and counterclockwise to the distractor location, respectively. (B) The same choice preference data, overlaid with the best-fit cumulative Gaussian function (see Table 1). (C) Distractor-value modulation (high − low distractor value) of the standard deviation (or sigma) and the mean (or mu) of the cumulative Gaussian function that explains choice preference in (B) (also see Table 1). Overall, we observed no distractor-value modulation on choice preference functions: sigma and mu did not change with distractor value in trials in which the current distractor was previously selected or unselected. (D) Unlike choice preference data, we observed a robust distractor-value modulation on RTs. The RT effect was significant only for trials in which the distractor was previously selected. Black *** shows a significant distractor-value modulation compared to 0 with *p* < 0.001 (2-tailed; resampling test). Red * shows a significant difference between trials in which the current distractors were previously selected and unselected with *p* < 0.05 (1-tailed). All error bars show ±1 SEM. CW, clockwise; CCW, counterclockwise; n.s., no significant difference; RT, response time.

Overall, subjects selected higher-valued targets more often than lower-valued targets (Fig 2A, *p* ≤ 1 × 10^−6^, 2-tailed, resampling test). This indicates that subjects were able to learn the values assigned to the different colors. Next, we fit the choice preference data as a function of differential target value with a cumulative Gaussian function (Fig 2B; see a similar fitting procedure in [6]). We found no effect of distractor value (high − low distractor value) on the standard deviation (or sigma) and the mean (or mu) of the cumulative Gaussian function on trials in which the current distractors were previously selected (*p* = 0.9420 and 0.0784 for sigma and mu, respectively, 2-tailed) or unselected (Fig 2B; *p* = 0.5637and 0.8206 for sigma and mu, respectively, 2-tailed). Since we used a 2-parameter cumulative Gaussian model to fit 3 data points for each experimental condition, we conducted an additional analysis in which we used the reduced model that only varied sigma to ensure the reliability of the result. First, a nested F test showed that this reduced model performed as well as the full model in which both sigma and mu were optimized, suggesting that this reduced model is more parsimonious than the full model (F[4, 3] = 0.9643, *p* = 0.4206). Second, we found that fitting the data with this reduced model yielded consistent results in which there was no significant distractor-value modulation on the sigma value for either selected (*p* = 0.9428) or unselected condition (and *p* = 0.5365). The null distractor-value effect in the choice preference data is inconsistent with our previous study using a variant of this value-based learning task, in which we found robust distractor-value modulations on the sigma value of the choice preference function [6]. We think this is due to the fact that in the current design, the value assignment for each color changed much more frequently than the task design used in our previous study (every 8 versus 36 trials) [6]. That said, the null distractor effect on the choice preference data is also consistent with a large body of literature demonstrating smaller and more variable distractor-value effects on task accuracy [11,56,57].

Although there was no distractor-value modulation on the choice preference data, RTs differed significantly across different distractor types (Table 1). We observed a significant effect of distractor value (high − low distractor value) on RTs on trials in which the current distractor was previously selected (Fig 2D; *p* ≤ 1 × 10^−6^, 2-tailed). However, there was no distractor-value modulation on trials in which the current distractors were previously unselected (*p* = 0.2756, 2-tailed). Moreover, the magnitude of the distractor-value modulation was significantly higher for the current distractor that was previously selected versus unselected (*p* = 0.0102, 1-tailed). These RT results show that the distractor value captures attention, leading to a relative decrease in the speed with which subjects processed task-relevant targets [5–8,13–17].

**Table 1:** Cumulative Gaussian best-fit variables describing choice preference data and RTs for different distractor types shown in Fig 2.

Behavioral Measurements	Distractor Types: Distractor Value and Selection History (Mean ± SEM)			
	Low and Unselected	High and Unselected	Low and Selected	High and Selected
Sigma	23.99 ± 8.01	29.87 ± 9.48	32.66 ± 7.09	33.56 ± 7.81
Mu	0.85 ± 2.41	0.17 ± 2.80	1.31 ± 1.58	1.46 ± 1.13
RTs (ms)	600 ± 20	612 ± 15	592 ± 18	643 ± 19

Abbreviation: RT, response time

### The reward history of distractors modulates neural representations in early visual cortex

**Fig 3:**
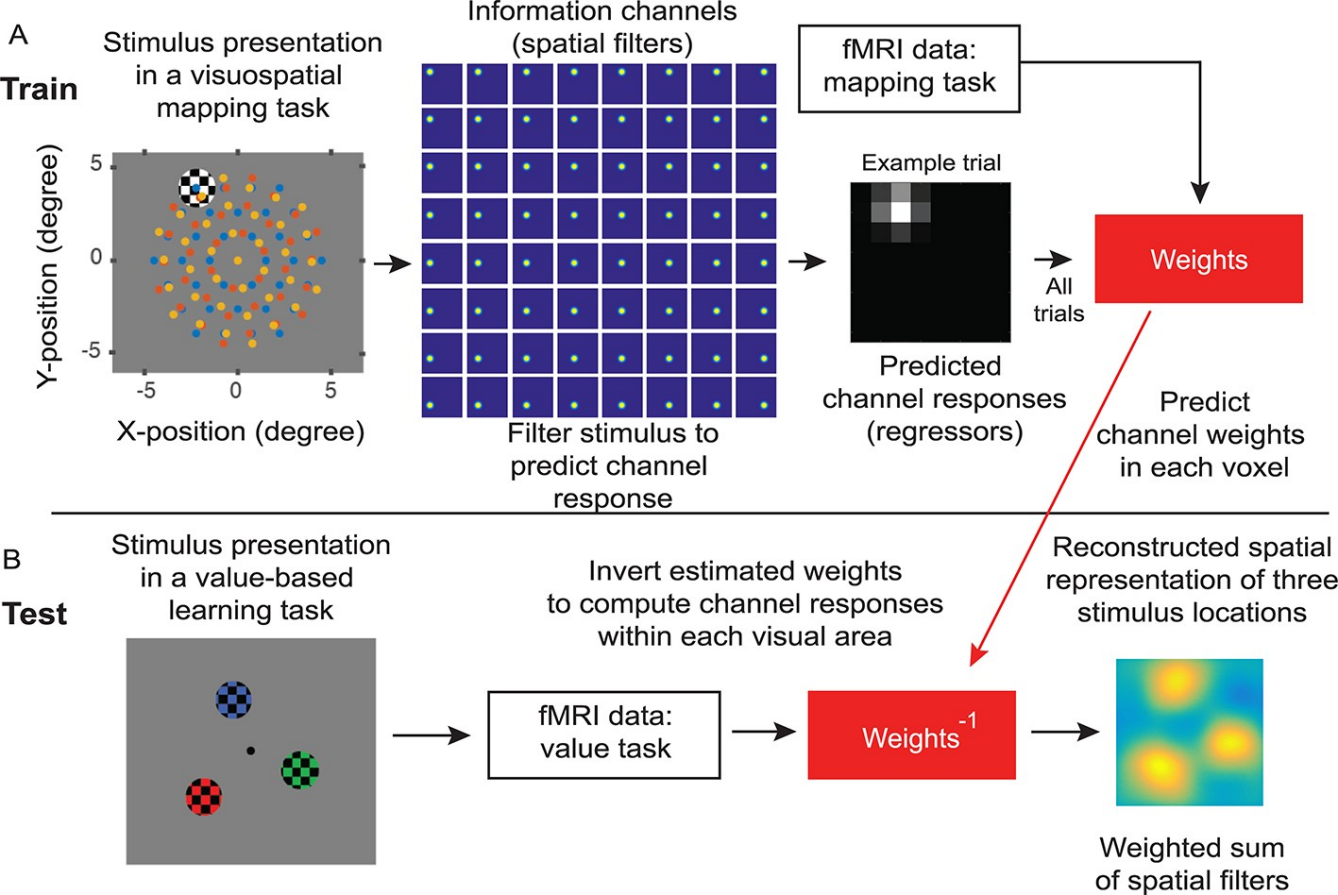
Quantifying stimulus representations with an IEM. (A) The IEM was trained using fMRI data from the visuospatial mapping task, in which flickering-checkerboard mapping stimuli were randomly presented at each of 111 locations (center locations shown in blue, red, and yellow dots in the first panels; these dots were not physically presented to participants). We filtered individual stimulus locations using 64 Gaussian-like spatial filters to predict channel responses for each trial. We then use the predicted channel responses and fMRI data of all trials to predict channel weights for each voxel within each visual area. (B) The IEM was tested using fMRI data from the value-based learning task (an independent data set). We inverted the estimated channel weights to compute channel responses within each visual area, resulting in a spatial reconstruction centered at 3 stimulus locations in the value-based learning task. fMRI, functional magnetic resonance imaging; IEM, inverted encoding model.

**Fig 4:**
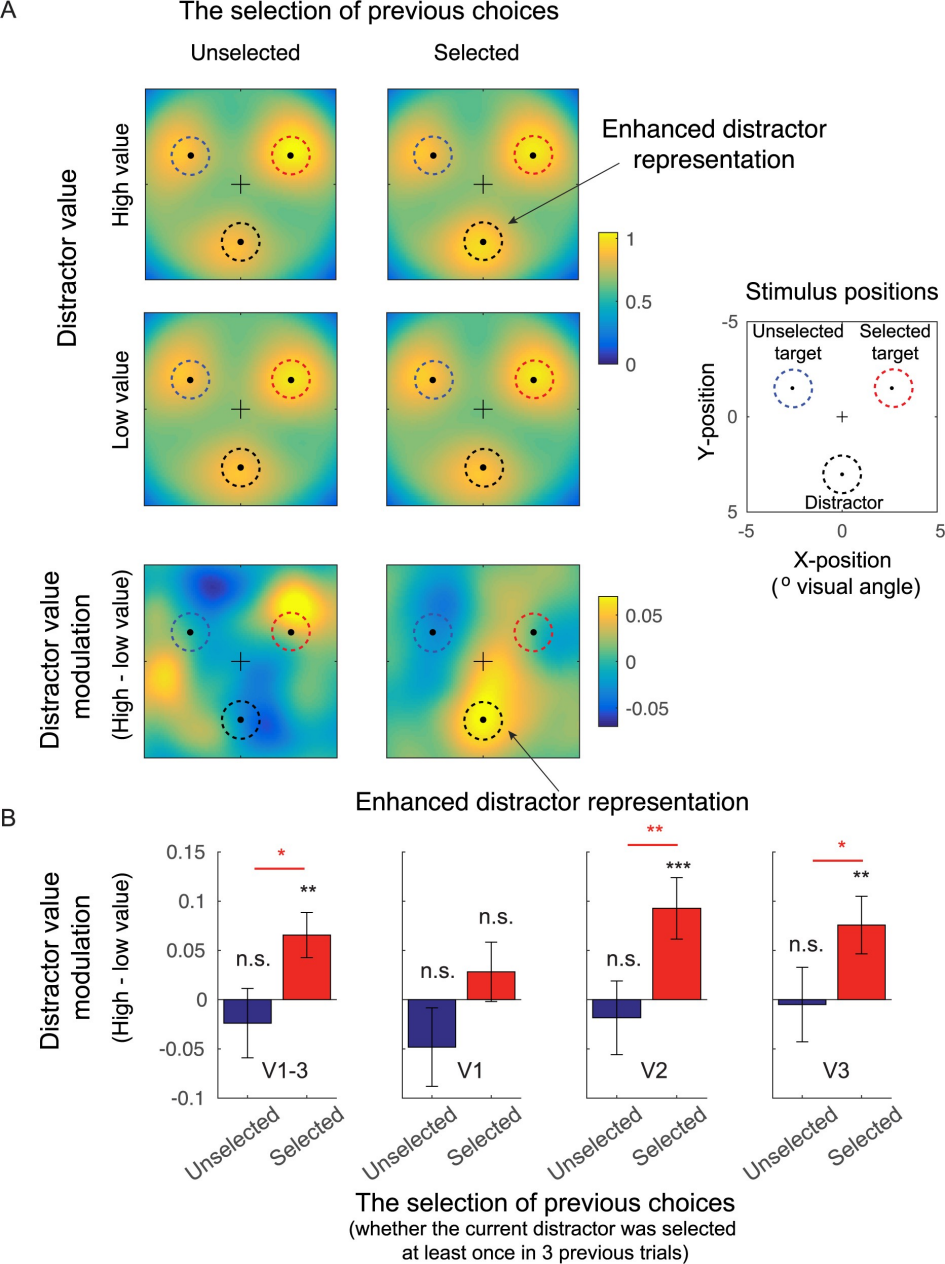
Distractor value boosted the activation of distractor representations in early visual cortex. (A) Averaged spatial reconstructions of the selected target, unselected target, and distractor based on fMRI activation patterns in early visual areas (collapsed across V1–V3). The data were sorted based on the distractor value (high and low distractor value) and the selection of previous choices (whether the current distractor was previously selected at least once in 3 prior trials: selected and unselected; also see Materials and methods). Before averaging, reconstructions were rotated so that the positions of each respective stimulus type were in register across subjects. In each color plot, a black dot marks the location of the central fixation, and 3 surrounding dots at 30°, 150°, and 270° polar angle indicate the centers of the selected target, unselected target, and distractor locations, respectively. The bottom panels show difference plots between high- and low-distractor-value conditions. (B) The distractor-value modulation (high − low distractor value) from the reconstruction activation (averaged across black dashed circles in [A]). Overall, we found significant distractor-value modulations in extrastriate visual areas V2 and V3, only in trials in which the current distractor was previously selected. Black ** and *** show significant distractor-value modulations compared to 0 with *p* < 0.01 and *p* < 0.001 (2-tailed). Red * and ** show a significant difference between trials in which the current distractors were previously selected and unselected with *p* < 0.05 and *p* < 0.01 (1-tailed). The statistics computed for different visual areas were corrected using the Holm-Bonferroni method. All error bars show ± 1 SEM. Blue, red, and black dashed circles in (A) represent the spatial extents of unselected targets, selected targets, and distractors, respectively. fMRI, functional magnetic resonance imaging; n.s., no significant difference.

To examine the influence of the distractor value on spatially specific distractor- and target-related neural representations in early visual cortex, we employed a multivariate analysis of fMRI data—an IEM (Materials and methods; Fig 3) [20,50,52,58]. The IEM exploits the spatial tuning of neuronal populations in visual cortex to reconstruct model-based representations of target and distractor stimuli based on population-level activity measured via fMRI. As expected, we found that these reconstructions peaked at the center of each of the 3 locations (Fig 4A; sorted as unselected target, selected target, and distractor). Qualitatively, the reconstructed activation at the distractor location was highest when the distractor colors matched the target colors that had been selected and rewarded with a higher value in the previous trials (i.e., the high-valued and previously selected distractor, the top right of the Fig 4A), compared to all the other distractor types.

To quantify this effect, we computed the mean activation level in the reconstructed stimulus representations over the space occupied by the distractors (Fig 4A, see Materials and methods; also see [20]). Then, we used a nonparametric resampling method (i.e., resampling subjects with replacement) to evaluate the impact of distractor value (high versus low distractor values) on the mean activation of the distractor representation. We did this separately for trials in which the current distractor had been previously selected or unselected in preceding trials to determine whether distractor-value modulations depended on the selection history associated with the color of the distractor.

First, we analyzed the data averaged across V1–V3 (Fig 4B). We found a significant distractor-value modulation (high > low value) for the distractor that was previously selected (*p* = 1 × 10^−3^, 2-tailed) but a null result for the distractor that was previously unselected (*p* = 0.4956, 2-tailed, resampling test). We directly evaluated this effect and found that selection history significantly increased distractor-value modulation (*p* = 0.0243, 1-tailed, resampling test). We then repeated these tests separately for individual visual areas. We found significant distractor-value modulations for the previously selected distractor in extrastriate visual areas V2 and V3 (*p* = 0.0011 and *p* = 0.0052, passing the Holm-Bonferroni-corrected thresholds of 0.0167 and 0.025, respectively, 2-tailed) but not in the primary visual cortex V1 (*p* = 0.3318, 2-tailed). In V2 and V3, we confirmed that selection history had a significant effect on distractor-value modulation (*p* = 0.0086 and *p* = 0.0374, respectively, 1-tailed). Similar to the data averaged across V1–V3, there was no significant distractor-value modulation for the previously unselected distractors in any visual area (*p* = 0.2031, *p* = 0.6263, and *p* = 0.9230, for V1, V2, and V3, respectively, 2-tailed). In sum, we used an IEM to evaluate spatially specific representations of behaviorally irrelevant stimuli with an associated reward history. We found that the value associated with irrelevant visual features is encoded in spatially specific activation in early visual areas V2 and V3.

To address whether the distractor-value modulations were driven by knowledge about the value associated with a given color, we split trials into early and late phases based on trial position relative to the start of each miniblock (in which the value–color assignments changed). If value learning matters, we should see robust distractor-value modulations only in the late phase but not in the early phase of each miniblock. Consistent with this prediction, we found a significant main effect of learning on distractor-value modulations in early visual cortex (late > early phases; *p* = 0.0167, 2-tailed) (see S1 Fig). This learning effect was driven by a significant distractor-value modulation in the late phase and only on trials in which the colors of the distractors matched the colors of the previously selected targets (i.e., selected: *p* = 0.0038, passing a Holm-Bonferroni threshold of 0.0125, 2-tailed). There was no significant distractor-value modulation in the late phase of the miniblocks on trials in which the color of the distractors did not match the color of the previously selected targets (i.e., unselected: *p* = 0.3844, 2-tailed). Importantly, there was also no significant distractor-value modulation in the early phase for either of the two distractor types (*p* = 0.1976 and 0.1800 for selected and unselected, respectively, 2-tailed). The null result for the early phase also speaks against the contribution of reward-based priming; as priming effects should rely only on the most recent choices, we should have seen distractor-value modulations regardless of the amount of learning that subjects acquired in the miniblock.

To further test the possibility of priming effects influencing our results, we conducted an auxiliary analysis in which we sorted trials by coding whether the current distractor was selected immediately in the previous trial or was selected at least once in the 2 or 3 previous trials. To ensure that trials included in these different data-sorting approaches came from a similar range of trial positions relative to the start of each miniblock, we only included trials between the fourth and the eighth (i.e., the last) trials of each miniblock. We found that distractors that were selected on the immediately preceding trial did not induce a significant distractor-value modulation in the data (collapsed across V1–V3, *p* = 0.1367, 2-tailed). This suggests that distractor-value modulations were unlikely to be driven entirely by reward-mediated priming effects (S2 Fig, left). However, when we sorted trials based on past selections over the previous 2 and 3 trials, we observed a significant distractor-value modulation (*p* = 0.013 and *p* < 0.001 for the data sorted based on the 2 and 3 previous trials passing Holm-Bonferroni thresholds of 0.025 and 0.0167, respectively). Since these two data sorting approaches included trials in which the same color targets could be selected more than once, these results emphasize the importance of learning the reward-color contingencies in producing value-driven modulations as opposed to reward-mediated priming effects. Note that qualitatively similar, albeit weaker, results were observed in individual visual areas V1–V3 (see S2 Fig).

### Target selection and target value are encoded in early visual cortex

**Fig 5:**
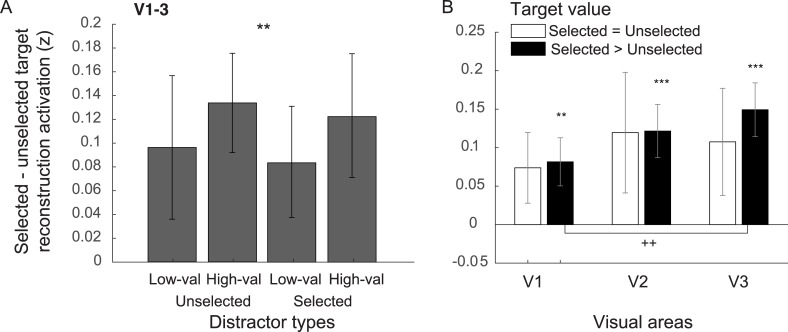
Target-selection modulations in early visual areas. (A) The difference between the selected and unselected target reconstruction activation for different target types. The activation values were obtained from averaging the reconstruction activation over circular spaces spanning the spatial extents of target stimuli (red and blue dashed circles in Fig 4A). The data in (A) were collapsed across visual areas. (B) The same data as (A) but plotted separately for different target-value conditions and for different visual areas. ** and *** indicate significant target-selection modulations compared to 0 with *p* < 0.01 and < 0.001, respectively (2-tailed). ^++^Significant difference across visual areas V1 and V3. Statistics in (B) were corrected for multiple comparisons with the Holm-Bonferroni method. All subfigures are plotted with ±1 SEM. val, valued.

As shown in Fig 3A, stimulus representations were generally higher for selected targets compared to unselected targets. To quantify this effect, we computed the mean activation level in the reconstructed stimulus representations over the space occupied by the selected and unselected targets (Fig 5A). For the data collapsed across V1–V3, we observed a significant target selection modulation (selected > unselected targets: *p* = 0.0011 for data collapsed across distractor types; *p* = 0.0642, 0.0003, 0.0228, and 0.0022 for low-valued and unselected, high-valued and unselected, low-valued and selected, and high-valued and selected distractors, with the Holm-Bonferroni-corrected thresholds of 0.05, 0.0125, 0.025, and 0.0167, respectively, 2-tailed). These target-selection modulations were significant in all visual areas (*p* = 0.0189, 4.600 × 10^−4^, and 5.600 × 10^−4^, V1, V2, and V3, respectively; Holm-Bonferroni-corrected, 2-tailed).

Next, we evaluated the impact of distractor value on the differential activity between selected and unselected targets. We found no influence of distractor value on target representations (high- versus low-valued distractors) on trials in which the current distractor was previously selected (*p* = 0.2303, 2-tailed) or on trials in which the current distractor was unselected (*p* = 0.4463, 2-tailed). Similar null results were also observed when the data were analyzed separately in V1, V2, and V3 (*p* = 0.1639–0.8710 and 0.0744–0.9419 for the selected and unselected conditions, 2-tailed). These are consistent with the null distractor-value effects on the choice preference data (Fig 2A and 2B).

Previous studies have reported that the relative value of targets is encoded in early visual cortex [23–25]. To test this, we analyzed the target-selection modulation data both when the selected and unselected targets had the same value (i.e., selected = unselected targets) and when the selected target had a higher value compared to the unselected target (i.e., selected > unselected targets). As shown in Fig 5B, we found significant target-selection modulations only when the selected targets had a higher value compared to the unselected targets in all visual areas (*p* = 0.0055, 4 × 10^−6^, and 1 × 10^−6^, passing the Holm-Bonferroni-corrected thresholds of 0.0125, 0.0100, and 0.0083 for V1, V2, and V3, respectively, 2-tailed), but no significant target modulations when selected and unselected targets had the same value (*p* = 0.0437–0.0756, which did not pass the Holm-Bonferroni-corrected threshold of 0.0167, 2-tailed). In addition, on trials in which participants selected the higher-valued target, the target-selection effect was significantly stronger in V3 than in V1 (*p* = 0.0021, passing the Holm-Bonferroni-corrected threshold of 0.0167, 2-tailed). However, there was not a significant difference between V3 and V2 (*p* = 0.1165, 2-tailed) or between V2 and V1 (*p* = 0.1274, 2-tailed). Taken together with the previous section, our results show that the encoding of target value and distractor value can occur in parallel in early areas of visual cortex.

## Discussion

Visual stimuli that are not physically salient but are paired with high-reward values are known to automatically capture attention, even when those stimuli are behaviorally irrelevant and unactionable [5–9]. Although a recent study reported that neural responses associated with distractors scale with the learned value [35], it is unclear whether these modulations were tied specifically to the location of the distractor and whether distractor response modulations led to attenuated target responses. Using a multivariate spatial reconstruction analysis of fMRI data, we show here that retinotopically organized regions in extrastriate visual areas V2 and V3 are modulated by the reward history of irrelevant visual stimuli. Importantly, the spatial reconstructions of these stimuli indicate that reward-based modulations occur precisely at the location of the distractor and that there is little associated impact on responses to simultaneously presented targets. Taken together, our results suggest that value-driven attentional capture may begin with early value-based modulation of sensory responses evoked by the distractor.

Importantly, these attentional capture effects on task-irrelevant distractors suggest that value-driven attention is not simply due to an increase in the non-stimulus-specific arousal levels induced by the reinforcement process, consistent with the finding that perceptual learning can occur even when task-irrelevant subthreshold stimuli are paired with rewards [36,59]. The fact that we observed value-based modulations that were spatially specific to the distractor location strongly supports this argument.

At first glance, our results seem to contradict several recent studies that observed a reward-based suppression of neural representations associated with distractors in sensory cortices [38–40,60]. However, in many of these studies, the reward manipulation was not specifically tied to the distractor, and distractor suppression was inferred based on modulations of neural responses related to the task-relevant targets [39,40,60]. Thus, these recent results are actually in line with the current data, in which the reconstruction activation of selected targets was higher than unselected targets and low-valued distractors. That said, another recent study reported that a high-valued distractor induced weaker neural representations in early visual cortex compared to the low-valued distractor [38]. However, they found that this was true only when the distractor was physically more salient than the target in a perceptually demanding task [38]. They reasoned that the high sensory competition between low-salience targets and high-salience distractors required top-down attentional suppression of the high-valued distractors [38]. However, this was not the case in the current experiment, in which all stimuli were suprathreshold and matched for luminance. Thus, in the context of our experimental design, we did not find evidence for distractor suppression at either the behavioral or neural level.

In the present study, we showed that an association between reward and color can induce neural modulations in early visual areas V1–V3. This is somewhat surprising, given evidence that neurons in higher visual areas (such as V4, V8, VO1, and inferior temporal cortex) are selectively tuned to chromatic information and responsible for processing color-based top-down modulations [48,58,61–65]. We suggest that value-based modulations in early visual areas may reflect top-down feedback signals from these higher visual areas, where the association between color and reward might be computed. Related to this idea, we found significant distractor-value modulations only in extrastriate visual cortex but not in V1, which may reflect a reentrant signal backpropagated to earlier visual areas. The more robust effects in higher visual areas were also observed for the task-relevant target reconstructions, consistent with previous reports [20,43,50,52,66]. Overall, this pattern of data supports theoretical frameworks suggesting that visual cortex operates as a priority map that indexes the rank-ordered importance of different sensory inputs [20,23–25,39,40,50,52,67,68]. That said, the assumption that the color–reward association can only be computed in higher visual areas has to be considered with caution, because previous studies have found that reward learning can shape neural activity in early visual cortex [24,25,69], and others have also found that primary and extrastriate visual areas contain neuronal populations with an inhomogeneous spatial distribution of color selectivity [42,70].

It is possible that the voluntary allocation of feature-based attention could be one selection mechanism that is guided by learned value. For instance, as subjects learned the value associated with each color, they might have selectively attended to higher-valued colors as each miniblock progressed. Since feature-based attention has been shown to operate globally across the visual scene, the prioritization of higher-valued colors could in turn enhance the neural representations of visual stimuli rendered in those colors even when they appeared outside the focus of attention [70–78]. This global effect of feature-based attention could induce attentional capture to the distractor location when the feature of the distractor matches the prioritized feature in the top-down set—akin to classic contingent capture effects [79–81]. Indeed, there has been an on-going debate about whether value-driven attention should be considered as another form of top-down attention [9,82–86]. However, value-driven attention capture has many characteristics that are distinct from top-down attention [83,85–87]. For example, value-driven capture can happen involuntarily and can be detrimental to behavioral goals, two effects that were demonstrated via the effect of distractor value on RTs in the present study. Therefore, even though value-based modulations of distractor-related neural representations might involve some aspects of top-down feature-based attention, we believe that these modulations reflect involuntary value-driven attentional capture. Also note that although some subjects might choose to voluntarily search for a color previously associated with a high reward, this seems unlikely because this strategy would hurt overall performance and slow the process of evaluating the value of the two relevant targets, as the targets will not always be rendered in the color that the subject is searching for.

Recently, it has been suggested that value-driven attentional capture is valence-free because both monetary gain and loss could produce similar attentional capture effects on RTs [88]. Based on this result, one might speculate that value-based modulations on the distractor-related neural representations shown here could potentially be independent of valence. However, a recent study has suggested that the value-based modulations of object-based stimulus representations in object-selective cortex depend on valence [60]. That said, as discussed earlier, in this study monetary gain and loss were not tied to the distractor stimulus itself [60]; thus, it is hard to conclude from this study alone whether value-based modulations on the distractor-related neural representations are valence-free. Future studies could employ our experimental approach in combination with the manipulation of monetary gain and loss to address this question further.

In summary, we demonstrate that the learned value of irrelevant distractors automatically captures attention and that this interferes with the processing of relevant visual information. This value-driven attentional capture results in increased RTs and heightened distractor representations in retinotopically organized areas of extrastriate visual cortex. Together, our findings suggest that value-driven attentional capture begins with early sensory modulations of distractor representations in visual cortex. Moreover, the modulations of both relevant targets and irrelevant distractors support a recent reframing of the classic dichotomy between bottom-up and top-down biasing factors in favor of a trichotomy that emphasizes a crucial role of value learning on the processing of relevant and irrelevant visual information [9].

## Materials and methods

### Ethics statement

Participants were recruited from the University of California, San Diego (UCSD), community, and all participants provided written informed consent as required by the local institutional review board at UCSD (IRB# 081318).

### Participants

Sixteen neurologically healthy human observers with normal color vision and normal or corrected-to-normal acuity participated in the present study. They then completed 1 scanning session of the main experiment and 1 or 2 sessions of retinotopic mapping scans. Participants were compensated $20 per hour in the scanner, with additional monetary rewards that scaled with their behavioral performance in the value-based learning task (mean $13.13, SD 0.74). Data from 1 subject were excluded because of excessive movement artifacts during the retinotopy scans (>3 mm movement in more than half of the scans), leaving a total of 15 participants in the final analysis (age range 20–34 years old, mean age = 24.6 years ± 4.29 SD).

### Stimuli and tasks

Visual stimuli were rear-projected onto on a 115-cm-wide flat screen placed approximately 440 cm from the participant’s eyes at the foot of the scanner bore using an LCD projector (1,024 × 768, 60 Hz, with a gray background, luminance = 8.68 cd/m^2^). The behavioral paradigms were programmed and presented via a laptop running Windows XP using MATLAB (Mathworks, Natick, MA, United States) and the Psychophysics Toolbox [89,90].

### Value-based decision-making task

We adopted a value-based decision-making task that we recently used to show a robust effect of distractor reward history on behavior [6]. Each block started with an instruction period telling participants the locations of the two targets and the location of the irrelevant distractor. The position of each stimulus was indicated by different letter strings located inside three 3 circular placeholders equally spaced from one another (120° polar angle apart with an eccentricity of 3.02° visual angle; Fig 1). The placeholders remained visible for the entire run so that participants knew the precise target and distractor locations. The instruction period was followed by experimental trials in which 3 physically isoluminant checkerboard stimuli of different colors were presented (black paired with red, green, and blue; radius of 1.01° visual angle; spatial frequency of 1.98 cycles per degree visual angle). The stimuli were flickered on–off at 7.5 Hz for 1 s.

Participants were instructed to choose 1 of the 2 targets to maximize their reward and were told that the reward value associated with each color changed across the course of the scan. The reward values associated with each stimulus color were changed every 8 trials (a miniblock). Subjects were not explicitly informed about the length of this miniblock, but they were told that reward–color associations would change dynamically across a small chunk of trials. All 8 possible combinations of the 3 colors and 2 reward values (1 and 9 cents) were presented in each miniblock. The color assignments to each target and distractor stimulus were also counterbalanced within each miniblock. Trial order was pseudorandomized so that the colors of the visual stimuli at 3 stimulus locations swapped in an unpredictable fashion. The assignment of different values to each color was also randomized so that changes in color–reward associations were unpredictable.

Participants were instructed to choose 1 of the 2 targets using 2 fingers on the right hand, as indicated in a diagram displayed before the run started (Fig 1). Importantly, the distractor could never be chosen and was thus choice-irrelevant. After a 1.25-s delay following the offset of the stimulus array, participants received visual feedback indicating the value associated with the chosen target color (“1” or “9”; feedback duration = 0.25 s). If a response was not given before the stimulus offset, they would receive a letter “M” (“miss”) to indicate that no reward was earned on that trial. In a random 20% of trials, rewards were withheld to encourage participants to explore and learn the value of each color (done independently for each of the two targets). In these trials, “0” cents were given, indicating that participants received no reward. The feedback period was followed by a blank intertrial interval with a central fixation for 1.5 s.

Participants completed 6 total blocks with the distractor location remaining stable for 2 consecutive blocks to ensure that participants knew the exact position of the distractor stimulus. Across all blocks the distractor location was counterbalanced across the 3 possible stimulus positions. Each block lasted 4 min 57 s and contained 48 experimental trials and 20 pseudorandomly interleaved null trials. There was a blank period of 9 s at the end of each block. We counterbalanced stimulus configurations across participants to ensure our results were not influenced by any spatial bias. To sample data from the entire circular space across subjects, the stimulus arrays were rotated by 30° polar angle to form 4 configurations (15°-135°-255°, 45°-165°-285°, 75°-195°-315°, and 105°-225°-345°), and these 4 configurations were counterbalanced across subjects. Each subject viewed 1 of these 4 configurations for their entire scanning session.

### Visuospatial mapping task

Participants also completed 4–7 blocks of a visuospatial mapping task (1 completed 4 blocks, 1 completed 7 blocks, and the rest completed 6 blocks). The data from this task were then used as an independent data set to train an IEM that was used to reconstruct spatial representations of the targets and distractors in the value-based learning task (see the fMRI analysis section below for more details). Participants were instructed to fixate centrally and to covertly attend to a checkerboard stimulus rendered at 100% Michelson contrast that pseudorandomly appeared at different locations on the screen (3-s duration; the same size, spatial frequency, and flicker frequency as the stimulus in the value-based learning task). The participant’s task was to detect a rare and brief dimming in contrast (19.57% target trials; 0.5-s duration; occurring between 0.5 and 2 s after stimulus onset). On each trial, the checkerboard stimulus was presented at 1 of 37 locations on a triangular grid (1.50° visual angle between vertices), covering a visual space that overlapped with the stimulus locations in the value-based learning task (the first panel in Fig 3A). To smoothly cover the entire circular space, we randomly rotated the entire triangular grid around its center by 0°, 20°, or 40° polar angle across different runs (blue, yellow, and red dots in the first panel in Fig 3A), so there were 111 different stimulus locations in total (see similar methods in [20]). On each run, there were a total of 37 nontargets (1 repeat per location) and 9 targets. Target locations were pseudorandomly drawn from the 37 locations (never repeated within each block). The magnitude of the contrast change was adjusted across trials so that accuracy was at approximately 76% (mean hit = 77.95%, SD = 12.23%). Each stimulus presentation was followed by an intertrial interval of 2–5 s (uniformly distributed). We pseudorandomly interleaved 10 null trials and included a blank period of 8.2 s at the end of the block. Each block lasted 6.28 min.

### Behavioral analysis

We first sorted trials from the main value-based decision-making task based on target selection (i.e., target type: selected and unselected), target value (low and high value), distractor value based on previous target rewards associated with the color of the distractor (low and high value), and selection history (i.e., whether the distractor was previously unselected or selected at least once in 3 preceding trials). We chose the 3 previous trials as the analysis window because it yielded the most balanced number of trials between individual conditions. Note that because of the boundary between miniblocks (every 8 trials in which value–color assignments were the same), we could only go back 1 and 2 trials for the second and third trials, respectively. We excluded data from the first trial of every 8 trials in each miniblock to reduce the spillover effect from different sets of value–color assignments.

Next, we examined subjects’ choice preference. To do so, we labeled targets located clockwise (CW) and counterclockwise (CCW) to the distractor CW and CCW targets and computed the probability that participants chose CW over CCW targets and plotted as a function of CW target value and CCW target value (Fig 2A). Next, we plotted the choices as a function of differential target value (CW − CCW) separately for different distractor values and fit individual subjects’ data with the cumulative Gaussian function (Fig 2B). Specifically, we estimated the mean (or mu) and the standard deviation (or sigma) of the cumulative Gaussian function that best fit the choice preference data derived from different distractor values (see Table 1 for mean and SEM; [6]). To test distractor-value modulations on these values, we computed the bootstrap distribution of the difference in these values between the high- and low-distractor-value conditions (i.e., resampling subjects with replacement for 100,000 iterations) and calculated the percentage of values in this distribution that were larger or smaller than 0 to yield a 2-tailed *p*-value. We performed this statistical analysis separately for previously selected and unselected distractors (see above). Note that we use the 2-parameter cumulative Gaussian model that varied sigma and mu to be consistent with a previous study by our group, even though there were only 3 data points for each experimental condition [6]. To ensure that the fitting procedure provided reliable results, we also used a variant of the model in which we only optimized sigma for each experimental condition while fixing mu at 0. We then did the same resampling analysis on sigma obtained from this reduced model to test the effect of distractor value. We compared the performance of this reduced model with the full model (above) using the nested F test (see [91–93]). To do so, we compared the R^2^ values between these two models using the following equation:

$$F({Df}_{1},{Df}_{2})=\left(\frac{R_{full}^{2}-R_{red}^{2}}{{Df}_{1}}\right)/\left(\frac{1-R_{red}^{2}}{{Df}_{2}}\right),$$ (1)

where *R*_*full*_^*2*^ and *R*_*red*_^*2*^ were obtained from the best fits of the full and reduced models, respectively. *Df*_*1*_ is the number of values in the full model (8 free parameters: 4 sigma values and 4 mu values for high/low-valued previously selected/unselected distractors) minus the number of the values in the reduced model (4 sigma values for different distractor types: high/low-valued previously selected/unselected distractors). *Df*_*2*_ is the number of observations (3 differential target values × 4 distractor types) minus the number of the free parameters in the full model minus 1. The *F* distribution was then used to estimate the probability that the full model differed significantly from the reduced model.

Finally, we examined the effect of distractor value on RTs. First, we computed the mean RTs across different distractor values for individual subjects. Then, we computed the bootstrap distribution of the RT difference between the high and low distractor-value conditions (i.e., resampling subjects with replacement for 100,000 iterations) and calculated the percentage of values in this distribution that were larger or smaller than 0 (a 2-tailed *p*-value). We performed this statistical analysis separately for previously selected and unselected distractors. We then compared whether the effect of distractor value was significantly larger in the selected condition than in the unselected condition by a similar procedure that compared the two bootstrap distributions. Since we only observed significantly larger RT differences for previously selected targets, we knew the expected direction of the effect and therefore computed a 1-tailed *p*-value.

### fMRI analysis

#### fMRI acquisition

All MRI data were acquired on a GE 3T MR750 scanner at the Keck Center for Functional Magnetic Resonance Imaging (CFMRI) at UCSD. Unless otherwise specified, all data were collected using a 32-channel head coil (Nova Medical). We acquired functional data using a multiband echo-planar imaging (EPI) protocol (Stanford Simultaneous Multi-Slice sequence). We acquired 9 axial slices per band at a multiband factor of 8, for 72 total slices (2 × 2 × 2 mm^3^ voxel size; 800-ms TR; 35-ms TE; 35° flip angle; 104 × 104 cm matrix size). Prior to each functional scan, 16 TRs were acquired as reference images for image reconstruction. Raw k-space data were reconstructed into NIFTI format image files on internal servers using scripts provided by CFMRI. In each session, we also acquired forward and reverse phase-encoding blips to estimate the susceptibility off-resonance field [94]. This was used to correct EPI signal distortion using FSL topup [95,96], the results of which were submitted to further preprocessing stages described below. In each session, we also acquired an accelerated anatomical using parallel imaging (GE ASSET on a FSPGR T1-weighted sequence; 1 × 1 × 1 mm^3^ voxel size; 8,136-ms TR; 3,172-ms TE; 8° flip angle; 172 slices; 1-mm slice gap; 256 × 192 cm matrix size). This same-session anatomical was coregistered to the functional data. It was also coregistered to a high-resolution anatomical from the retinotopic mapping session(s).

#### Retinotopic mapping

To identify regions of interest (ROIs) in early visual cortex, we used a combination of retinotopic mapping methods. Individual participants completed meridian mapping (1–2 blocks of approximately 5 min), during which they saw flickering checkerboard “bowties” along the horizontal and vertical meridians while fixating centrally. They also completed several scans of a polar angle mapping task (4–6 blocks of about 6 min), during which participants covertly attended to a rotating checkerboard wedge and detected brief contrast changes (see details in [20,52]). We identified retinotopically organized regions of visual areas V1, V2, and V3 using a combination of retinotopic maps of visual field meridians and polar angle preferences for each voxel in these visual areas and concatenated left and right hemispheres as well as dorsal and ventral aspects of individual areas [97,98]. Visual area borders were drawn on an inflated cortical surface created from a high-resolution anatomical scan (FSPGR T1-weighted sequence; 1 × 1 × 1 mm^3^; 8,136-ms TR; 3,172-ms TE; 8° flip angle; 172 slices; 1-mm slice gap; 256 × 192 cm matrix size) collected with an 8-channel head coil.

#### fMRI data preprocessing

Analysis was performed in BrainVoyager 20.2 (Brain Innovation, Maastricht, the Netherlands) supplemented with custom analysis scripts written in MATLAB R2016a (Mathworks, Natick, MA, USA). Using the distortion-corrected images, we first performed slice-time correction, affine motion correction, and temporal high-pass filtering. Then, the functional data were coregistered to the same-session anatomical and transformed to Talairach space. Each voxel’s time course was *z*-scored within each run. We then built a design matrix with individual trial predictors convolved with a double-gamma HRF (peak = 5 s, undershoot peak = 15 s; response undershoot ratio = 6; response dispersion = 1; undershoot dispersion = 1). We also included a baseline predictor. This allowed us to calculate single-trial beta weights using a general linear model (GLM). These beta weights served as input to the IEM described in the next section.

#### IEM

In order to create model-based reconstructions of target and distractor stimuli in the value-based learning task from individual ROIs, we employed an IEM for retinotopic space (see Fig 3; also see [42,48–50,52]). First, we computed a spatial sensitivity profile (i.e., an encoding model) for each voxel, parameterized as a weighted sum of experimenter-defined information channels (i.e., spatial filters in the second panel of Fig 3A) using an independent training data set acquired from the visuospatial mapping task (using only the nontarget trials). Then, we inverted the encoding models across all voxels to compute weights on the spatial information channels and used these weights to transform the fMRI data from the value-based learning task into an activation score. This inversion step provides one means of assessing how much information is encoded about a target or distractor stimulus by aggregating modulations across the entire population of voxels in a given visual area.

More specifically, the activation of each voxel is modeled as the weighted sum of 64 bivariate Gaussian-like spatial information channels arrayed in an 8 × 8 rectangular grid (see the second panel of Fig 3). The filter centers were equally spaced by 1.43° visual angle with a full-width half-maximum of 2° visual angle). The Gaussian-like function of each filter is described by:

$$f(r)={\left(0.5+0.5\;\cos\frac{\pi r}{s}\right)}^{7}\mathrm{for}\;r<s;\;0\;\mathrm{otherwise},$$ (2)

where *r* is the distance from the filter center and *s* is a size value indicating the distance between filter centers at which the filter returns to 0. We set values greater than *s* to 0 (*s* = 5.0332), resulting in a smooth filter at each position along the grid [50].

We then defined the idealized response of the information channels for each given training trial. To do this, we multiplied a discretized version of the stimulus (*n* trials × *p* pixels) by the 64 channels defined by Eq 1 (*p* pixels × *k* channels). We then normalized this result so that the maximum channel response is 1. This is *C*_*1*_ in the following equation:

$$B_{1}=C_{1}W,$$ (3)

where *B*_*1*_ (*n* trials × *m* voxels) is the measured fMRI activity of each voxel during the visuospatial mapping task (i.e., beta weights, see fMRI preprocessing section), *C*_*1*_ (*n* trials × *k* channels) is the predicted response of each spatial filter (i.e., information channel normalized from 0 to 1), and *W* is a weight matrix (*k* channels × *m* voxels) that quantifies the contribution of each information channel to each voxel. Next, we used ordinary least-squares linear regression to solve for *W* with the following equation:

W^=(C1TC1)−1C1TB1 (4)

Here, W^ represents all estimated voxel sensitivity profiles, which we computed separately for each ROI. Next, we used W^ and the measured fMRI activity of each voxel (i.e., beta weights) during each trial of the value-based learning task to estimate the activation of each information channel using the following equation (see Fig 3B):

C^2=B2W^T(W^W^T)−1 (5)

Here, C^2 represents the estimated activation of each information channel (*n*_*2*_ trials × *k* channels), which gives rise to the observed activation pattern across all voxels within that ROI (*B*_*2*_, *n*_*2*_ trials × *m* voxels). To visualize and coregister trials across 3 stimulus locations, we computed spatial reconstructions by multiplying the spatial profile of each filter by the estimated activation level of the corresponding channel (i.e., computing a weighted sum; the last panel of Fig 3B). We rotated the center position of the spatial filters on each trial of individual participants such that the resulting 2D reconstructions of the target and distractor stimuli share common positions across trials and participants (CCW target, CW target, and distractor locations centered at 30°, 150°, and 270° polar angle, respectively; 3.02° visual angle from the center of the 2D reconstruction). Next, we sorted trials based on selected and unselected target values (low and high), the reward history of the distractor (low and high), and whether the current distractor had been selected or unselected in the 3 previous trials in the same way as we did for the behavioral analysis. Then, we flipped all spatial reconstructions left to right on trials in which the selected target location was on the left (150°) so that the unselected and selected targets always shared common locations on the left and right of the reconstruction, respectively (150° and 30°). This step did not change the position of the distractor, so it stayed at a 270° polar angle. Finally, we averaged the 2D reconstructions across trials with the same trial types for individual participants and then averaged those reconstructions across participants, resulting in the grand-average spatial reconstructions shown in Fig 4A.

#### fMRI statistical analysis

Following a previous approach [20,53], we extracted the reconstruction activation for each trial type in individual participants by averaging the data within the circular space spanning the entire area of individual stimuli. This was used as our “reconstruction activation” measure. Like the behavioral analyses, all statistical analyses were conducted by resampling relevant values from each subject with replacement for 100,000 iterations and comparing these values across resampling iterations

In the main analysis, we examined the distractor-value modulation on the distractor reconstruction activation for data averaged across V1–V3. To do so, we computed the bootstrap distribution of the difference of the distractor reconstruction activation between the high and low distractor-value conditions and calculated the percentage of values in this distribution that were larger or smaller than 0 (2-tailed). We performed this statistical analysis separately for trials in which the current distractor was previously selected and unselected in preceding trials to examine whether the distractor-value modulation depended on the selection of previous choices. We then compared whether the effect of distractor value was significantly larger in the selected condition than in the unselected condition by a similar procedure that compared the two bootstrap distributions (1-tailed to the known direction of the difference). We repeated the same statistical procedures for individual visual areas and corrected for multiple comparisons using the Holm-Bonferroni method [99].

In addition, we ran 2 auxiliary analyses to examine the influence of the knowledge about the value of a given color and the possible effect of value-mediated priming on the distractor-related representations, respectively. In the first analysis, we divided the distractor-related representation data described above into trials that occurred early or late in each miniblock (second through fifth and sixth through eighth trials, respectively). We then examined the main effect of learning on the distractor-value modulation. To do so, we computed the bootstrap distribution of the difference of the distractor reconstruction activation between the high and low distractor-value conditions for the early and late phase. Then, we calculated the percentage of values between the two phases that were larger or smaller than 0 (2-tailed). We also computed the bootstrap distribution of the difference of the distractor reconstruction activation between the high and low distractor-value conditions and calculated the percentage of values in this distribution that were larger or smaller than 0 (2-tailed). This was done separately for the early and late phases as well as for trials in which the distractors were selected and unselected in previous trials. The Holm-Bonferroni method was used to correct for multiple comparisons.

The second auxiliary analysis was similar to the main analysis except that the selection of previous choices was based on whether the current distractor was selected at least once in 1, 2, or 3 preceding trials. To ensure that trials included in these different data-sorting approaches came from a similar range of trial positions relative to the start of each miniblock, we only included trials from the fourth through eighth trials of each miniblock. We examined the distractor-value modulation on the distractor-related representation in each of these data-sorting approaches by computing the bootstrap distribution of the difference of the distractor reconstruction activation between the high and low distractor-value conditions and calculated the percentage of values in this distribution that were larger or smaller than 0 (2-tailed). The Holm-Bonferroni method was used to correct for multiple comparisons. The procedure was first performed in the data collapsed across V1–V3, and the same analysis was then performed in individual visual areas.

Next, we tested the target selection modulation on the target reconstruction activation for data averaged across V1–V3. To do so, we computed the bootstrap distribution of the difference between the selected and unselected target reconstruction activation and calculated the percentage of values in this distribution that were larger or smaller than 0 (2-tailed). We first performed this on the data collapsed across all distractor types. Then, we assessed the target selection modulations separately for individual distractor values and corrected for multiple comparisons using the Holm-Bonferroni method. Then, we tested for the distractor-value modulation on the target-selection modulation by computing the bootstrap distribution of the difference of the target-selection modulations between the high and low distractor-value conditions and computing the percentage of values in this distribution that were larger or smaller than 0 (2-tailed). This was done separately for trials in which the current distractor was previously unselected and selected in preceding trials. We repeated the same statistical procedures for individual visual areas and corrected for multiple comparisons using the Holm-Bonferroni method.

Finally, we tested whether target-selection modulations depended on the relative value difference between selected and unselected targets, as suggested by previous studies [23–25]. For each target-value condition (same versus different target values) and each visual area, we computed the bootstrap distribution of the difference between the selected and unselected target reconstruction activation and calculated the percentage of values in this distribution that were larger or smaller than 0 (2-tailed). Here, we also corrected for multiple comparisons across different target value conditions and different visual areas using the Holm-Bonferroni method (6 comparisons). Since we found more robust target-selection modulations in higher visual areas in trials in which the selected and unselected targets had different values, we further tested whether the target-selection modulation in V3 was higher than that in V1, whether the target modulation in V2 was higher than that in V1, and whether the target modulation in V2 was higher than that in V1. To do so, we compared the target-selection modulation distributions across these visual areas (1-tailed, due to the known direction of the difference) and corrected for multiple comparisons using the Holm-Bonferroni method.

## Supporting information

S1 Fig Distractor-related modulations in early visual cortex rely on knowledge of the value associated with the distractor color. Same as Fig 4B but plotted separately for trials that were early or late in each miniblock (and color–value assignments were constant across all trials in a given miniblock). We found a significant distractor-value effect only in the late phase and only on trials in which the current distractor was a selected target on at least 1 of the 3 prior trials. **Significant distractor-value modulation compared to 0 with *p* < 0.01, Bonferroni-corrected (2-tailed). All error bars show ±1 SEM.
(EPS)

S2 Fig Distractor-value modulations related to a current distractor that was a selected target on one of the previous 1, 2, and 3 trial(s), respectively. We only included the fourth through eighth trials of each miniblock to ensure that the data across different data-sorting approaches came from a similar range of trial positions relative to the start of each miniblock. No significant effect was found when sorting the data based on just the immediately preceding trial (i.e., 1 trial back). This speaks against the possibility that reward-mediated priming effects due to the immediately preceding target selection can account for all of the value-based modulations. However, significant distractor-value modulations were observed when the current distractor was selected at least once during the last 2 and 3 trials. ** and *** represent significant distractor-value modulations compared to 0 with *p* < 0.01 and < 0.001, Bonferroni-corrected (2-tailed). All error bars show ±1 SEM.
(EPS)

## Abbreviations

fMRI: functional magnetic resonance imaging
IEM: inverted encoding model
RT: response time
n.s.: no significant difference
